# Feeling like David or rather Don Quixote?—hello from a new editor in chief

**DOI:** 10.1007/s00709-024-01972-9

**Published:** 2024-08-02

**Authors:** Joern Bullerdiek

**Affiliations:** Rostock University Medical Center, Rostock, Germany

As the new Editor-in-Chief of the animal branch of *PROTOPLASMA* who has been appointed in January 2024, I would like to introduce myself and to share some of my thoughts with the readers of this journal.

I am a human geneticist working at the medical center of the university of Rostock in the “Upper East side” of Germany. During the nearly hundred years that *PROTOPLASMA* does exist, it is not the first time that one of the editors in chief is working in Rostock. Josef Spek (1895–1965), together with Friedl Weber one of the two founding editors of *PROTOPLASMA*, was editor in chief from 1926 until 1964. He became professor for zoology at the university of Rostock in 1947.

But returning back to present: Since many years my basic scientific interests are devoted to mechanisms of tumor development regarding both genetics as well as cell biology. In the past several years, I already served as a member of the editorial board of the journal. Thus, needless to say that I felt honored by his offer when I had been asked by previous Editor-in-Chief Reimar Stick to take over this year. So first of all, thanks to him, to Peter Nick, the editor in chief of the plant side, and the other colleagues for their confidence and also special thanks again to Reimar Stick for all he has done for the journal in the past years.

Nevertheless, even though *PROTOPLASMA*, as a journal, is a prestigious old lady looking forward to celebrate the 100th birthday in 2 years, times have become worse for science journals today and it is easy to predict they will remain difficult for the next years. As part of the scientific community and along with all journals, we are facing a number of overlapping crises as, e.g., the papermill crisis (Van Noorden 2023) and the replication crisis (Crow 2024) which both affect cornerstones of science.

Sometimes, I feel like switching between two figures from the literature. On bad days I see myself resembling Don Quixote with the papermills, eh, sorry, windmills standing in front of me. In contrast, on better days I am rather feeling like David waiting for Goliath, already with the sling in my hands and with a positive feeling but still urgently looking for the suitable stones. However, of course I am not alone. In both branches of *PROTOPLASMA*, we have people in the editorial board who are all eager to help keeping *PROTOPLASMA* a prestigious journal in cell biology. I am also proud to mention that, as to the animal branch of the journal, we had been able to convince a couple of young scientists to join us as new members of the editorial board. In the next months, they will introduce themselves with reviews covering their fields as well as editorials.

Please note that we shall broaden and revise aims and scope of the journal a little bit. Accordingly, we are asking also for papers, e.g., in the fields of transcriptomics and (spatial) proteomics (Lundberg and Borner 2019) as well as those addressing mechanisms involving non-coding RNAs and cell to cell communication via exosomes.

Last but not least, we are looking forward to receive many interesting manuscripts. Almost needless to say, I am convinced that during the next years the David’s days will clearly dominate over Don Quixote’s days.
